# Differentially expressed mRNAs, proteins and miRNAs associated to energy metabolism in skeletal muscle of beef cattle identified for low and high residual feed intake

**DOI:** 10.1186/s12864-019-5890-z

**Published:** 2019-06-17

**Authors:** Elisa B. Carvalho, Mateus P. Gionbelli, Rafael T. S. Rodrigues, Sarah F. M. Bonilha, Charles J. Newbold, Simone E. F. Guimarães, Walmir Silva, Lucas L. Verardo, Fabyano F. Silva, Edenio Detmann, Marcio S. Duarte

**Affiliations:** 1 Department of Animal Science, Universidade Federal de Lavras, Lavras, MG Brazil; 2 Department of Animal Science, Universidade Federal de Viçosa, Viçosa, MG Brazil; 3 Department of Veterinary Sciences, Universidade Federal do Vale do São Francisco, Petrolina, PE Brazil; 4 Centro APTA Bovinos de Corte, Instituto de Zootecnia, Sertãozinho, SP Brazil; 5 Scotland’s Rural College, Edinburgh, UK; 6 Department of Animal Science, Universidade Federal dos Vales do Jequitinhonha e Mucuri, Diamantina, MG Brazil

**Keywords:** Bovine, Feed efficiency, Muscle biology, Nellore, Proteomics

## Abstract

**Background:**

Feed efficiency is one of the most important parameters that affect beef production costs. The energy metabolism of skeletal muscle greatly contributes to variations in feed efficiency. However, information regarding differences in proteins involved in the energy metabolism of the skeletal muscle in beef cattle divergently identified for feed efficiency is scarce. In this study, we aimed to investigate energy metabolism of skeletal muscle of Nellore beef cattle, identified for low and high residual feed intake using a proteomics approach. We further assessed the expression of candidate microRNAs as a one of the possible mechanisms controlling the biosynthesis of the proteins involved in energy metabolism that were differentially abundant between high and low residual feed intake animals.

**Results:**

A greater abundance of 14–3-3 protein epsilon (*P* = 0.01) was observed in skeletal muscle of residual feed intake (RFI) high animals (RFI-High). Conversely, a greater abundance of Heat Shock Protein Beta 1 (*P* < 0.01) was observed in the skeletal muscle of RFI-Low cattle. A greater mRNA expression of *YWHAE*, which encodes the 14–3-3 protein epsilon, was also observed in the skeletal muscle of RFI-High animals (*P* = 0.01). A lower mRNA expression of *HSPB1*, which encodes the Heat Shock Protein Beta 1, was observed in the skeletal muscle of RFI-High animals (*P* = 0.01). The miR-665 was identified as a potential regulator of the 14–3-3 protein epsilon, and its expression was greater in RFI-Low animals (*P* < .001). A greater expression of miR-34a (*P* = 0.01) and miR-2899 (*P* < .001) was observed in the skeletal muscle of RFI-High animals, as both miRNAs were identified as potential regulators of HSPB1 expression.

**Conclusion:**

Our results show that Nellore cattle divergently identified for feed efficiency by RFI present changes in the abundance of proteins involved in energy expenditure in skeletal muscle. Moreover, our data point towards that miR-665, miR34a and miR-2899 are likely involved in controlling both 14-3-3 epsilon and HSPB1 proteins identified as differentially abundant in the skeletal muscle of RFI-High and RFI-Low Nellore cattle.

**Electronic supplementary material:**

The online version of this article (10.1186/s12864-019-5890-z) contains supplementary material, which is available to authorized users.

## Background

Feed efficiency is one of the most important parameters affecting beef production costs. Residual feed intake (RFI) has been suggested as a more suitable trait for animal selection than the feed conversion ratio [1], as it is phenotypically independent of growth rate and body weight in growing cattle [2–4]. Although RFI has been extensively used to evaluate feed efficiency [5], its physiological and/or metabolic bases remain unclear. According to Herd and Arthur [6], at least five major processes are involved in the variation of efficiency: feed intake, feed digestion, animal activity, thermoregulation, and energy metabolism.

The turnover of body proteins accounts for 30% of energy expenditure in cattle maintenance. The metabolism of skeletal muscle represent an important factor regulating feed efficiency, since this tissue, together with liver tissue, account for around two-thirds of the whole body protein turnover in mammals [7]. However, most studies regarding molecular mechanisms underlying differences in feed efficiency in beef cattle have focused their efforts on changes at the transcriptome level [8–12], while information regarding differences at the proteomic level is scarce. Additionally, studies in cattle have suggested the role of microRNAs (miRNA) on feed intake at the post-transcriptional level [13, 14] and should be considered in further studies.

The miRNAs are a class of noncoding RNAs expressed in several eukaryotic organisms [15]. They are about 20 nucleotides (nt) long and regulate translation by binding to the 3′ untranslated region of their targets genes [15, 16]. Important roles of miRNAs in the gene-regulatory of numerous eukaryotic lineages have been reported [17]. Moreover, there is evidences of miRNAs related with feed efficiency and energy metabolism in pigs [18] and cattle [14], highlighting the importance of miRNAs in energy metabolism and, consequently, changes in feed efficiency.

We have hypothesized that Nellore cattle divergently identified for feed efficiency by RFI differ in energy expenditure in skeletal muscle. Therefore, our objective was to investigate the skeletal muscle proteomic profile of beef cattle diverging on RFI, aiming to identify key proteins that indicates difference in energy expenditure. We further assessed the expression of miRNAs as one of the possible post-transcription mechanisms controlling the expression of these proteins.

## Methods

Care and handling of all experimental animals were conducted under protocols approved by the Ethics Committee on Animal Use of the *Instituto de Zootecnia* (CEUA/IZ; Protocol 213–15)*,* Nova Odessa, SP*,* Brazil, and in accordance with guidelines of State Law No. 11.977 of the State of São Paulo, Brazil.

### Animals and experimental diet

A contemporary group of 129 young Nellore bulls [seven months of initial age and 239 ± 30.1 kg of initial body weight (BW)] were subjected to a growth period of 98 days receiving the same diet formulated to meet the requirements for 1 kg/d of BW gain (Table 1). Cattle were fed using a GrowSafe® automated feeding system (GrowSafe® Systems Ltd., Airdrie, Canada). The RFI (kg/day) was calculated after the growth period test using the following model:

$$ \mathrm{FI}={\upbeta}_0+{\upbeta}_{\mathrm{P}}\times {\mathrm{BW}}^{0.75}+{\upbeta}_{\mathrm{G}}\times \mathrm{ADG}+\upvarepsilon\ \left(\mathrm{RFI}\right), $$

where FI = estimated daily feed intake (kg/day), β_0_ = regression intercept, β_P_ = partial regression coefficient of FI on average metabolic weight, BW^0.75^ = metabolic body weight (kg), β_G_ = partial regression coefficient of FI on average daily gain (ADG, kg/day) and ε = residual error (RFI, kg/day).

**Table 1:** Ingredient and chemical composition of experimental diets

Item	Growth period	Finishing period
Ingredient, g/kg		
Corn silage	615	333
Brachiaria hay	33.0	17.0
Dry-ground corn	167	465
Soybean meal	163	163
Mineral premix^a^	18.0	13.0
Urea	3.60	6.00
Ammonium sulfate	0.40	4.00
Composition, g/kg of DM		
Dry matter, g/kg	496	636
Organic matter	937	946
Crude protein	137	134
Neutral detergent fiber	594	646
Ether extract	25.0	32.1

^a^ Provided (per kg of DM): 140 g of Ca, 137.2 g of Na, 12 g of S, 80 g of P, 4,500 mg of Zn, 1,400 mg of Mn, 11 mg of Ni, 1,600 mg of Cu, 210 mg of Co, 180 mg of I, 27 mg of Se, and 800 mg of F

From the 129 animals used in the growth period, a total of 18 Nellore bulls [nine with the lowest (− 1.18 ± 0.44) or highest (1.20 ± 0.46) estimated RFI] were selected, with a final age and BW of 22.5 ± 0.8 months and 401 ± 42 kg, respectively. Each selected animal was considered an experimental unit. All animals were confined in individual pens (measuring 4 × 2 m) with ad libitum access to water and diet. Feeding management was performed using the GrowSafe® automated feed system (Growsafe Systems Ltd., Airdrie, Alberta, Canada). The 18 selected animals were weighed, vaccinated, and dewormed at the beginning of the fishing period. Cattle were adapted to the diets, facilities, and management for 22 d and fed the finishing diet for a 103-d period. The finishing diet was composed of 333 g/kg corn silage, 17 g/kg brachiaria hay, 465 g/kg dry ground corn, 163 g/kg soybean meal, 6 g/kg urea, 4 g/kg ammonium sulfate, and 13 g/kg mineral mixture (dry matter basis), and were formulated to meet the requirements of 1.3 kg of daily gain with a target finish weight of 550 kg. Both RFI-High and RFI-Low groups were fed the same diet and kept under the same conditions throughout the finishing period. More details about the herd characterization, performance, carcass traits, and meat quality evaluations for these animals can be found in Fidelis et al. (2017) [19]. Details about the energy metabolism in the rumen epithelium of these animals can be found in a previous study [20] carried out by our research group.

### Animal slaughter and skeletal muscle tissue collection

At the end of the performance trial, bulls were transported to an experimental slaughterhouse abattoir for slaughter. Pre-harvest handling was conducted in accordance with good animal welfare practices, and slaughtering procedures followed strict guidelines established and regulated by the Sanitary and Industrial Inspection Regulation for Animal Origin Products [21]. Longissimus muscle was sampled immediately after exsanguination at the 12th rib of the right side of the carcass. Once collected, skeletal muscle tissue samples were immediately placed in liquid nitrogen then transported to the laboratory. Once samples arrived at the laboratory, they were powdered in liquid nitrogen using a mortar and pestle. Samples were then placed in a cryogenic tube and stored in liquid nitrogen until extraction of proteins, total RNA, and miRNA.

### Total protein extraction

Samples of skeletal muscle tissue (0.05 g) were homogenized using a polytron PT 3100 (Lucerne, Switzerland) on ice for 10s in 1 mL of lysis buffer containing: 7 M urea, 2 M thiourea, 4% 3–3 [(cholamidopropyl) dimethylammonium] -1- propanesulfonate (CHAPS), 1% dithiothreitol (DTT), 2% immobilized pH gradient (IPG) buffer (pH 3 to 10), 1 M phenylmethanesulfonyl fluoride (PMSF) and protease inhibitors. The supernatant was obtained after centrifugation at 10,000 x *g* for 30 min at 4 °C was used as the protein extract for later electrophoresis analysis. The total amount of proteins was quantified by Quick Start *Bradford* Protein *Assay (Bio-Rad,* Hercules, CA) using *Bovine serum albumin* (BSA) as a standard. The total proteins extract was separated by SDS-PAGE 10% gel by loading with 80 μg of protein per sample for quality control to check protein integrity.

### Two-dimensional electrophoresis

In the first dimension of electrophoresis, the Immobilized pH Gradient (IPG) strips (GE Healthcare Lifesciences, Uppsala, Sweden) of 24 cm, pH 3–10, were rehydrated overnight at room temperature in 450 μl of DeStreak rehydration solution (GE Healthcare Lifesciences, Uppsala, Sweden) and 2% IPG buffer pH 3–10, containing 1200 μg of protein. The, samples were then subjected to isoelectric focusing on an Ettan IPGphor3 system (GE Healthcare Lifesciences, Uppsala, Sweden) at 20 °C for the specific type of IPG strip (step and hold at 500 V for 1 h; gradient to 1000 V for 0.8kVh gradient to 10,000 V for 16.5 KVh, and step at 10000 V for 17.2 KVh), with a current limit of 50 mA/strip.

For the second dimension, strips were equilibrated in 1.5 M Tris–HCl (pH 8.8), 6 M urea, 2% sodium dodecyl sulfate (SDS), 30% glycerol, and 0.002% bromophenol blue buffer for 20 min with 1% dithiothreitol (DTT) followed by 20 min with 2.5% iodoacetamide. The strips were transferred to a 12.5% acrylamide gel and fixed with an agarose sealing solution. The SDS-polyacrylamide gel electrophoresis (SDS-PAGE) was performed in a vertical Ettan DALTsix system (GE Healthcare Lifesciences, Uppsala, Sweden) using a Laemmli running buffer at 1× concentration for the anode and 2× concentration for the cathode. The electrical current for electrophoresis was kept at 20 mA/gel with an initial voltage of 80 V for 45 min to allow proteins to migrate from the gel strip into the polyacrylamide gel. After this period, the voltage was increased to 500 V, using 40 mA/gel until the sample ran to the end of the gel. At the end of the run, gels were stained using a colloidal Coomassie Blue G-250 procedure, involving fixation in 10% acetic acid/40% ethanol overnight followed by addition of a solution containing 8% ammonium sulfate, 0.8% phosphoric acid, 0.08% Coomassie Blue G-250, 20% methanol for 72 h, and de-stained by a solution of acetic acid at 1%. Finally, gels were kept in a solution of 2% acetic acid until subsequent image analysis.

### Image analysis

The two-dimensional electrophoresis (2-DE) gels were scanned with ImageScanner III (GE Healthcare Bio-Sciences, Uppsala, Sweden), using the Lab Scan program (GE Healthcare Lifesciences, Uppsala, Sweden) and analyzed by using ImageMaster Platinum software (GE Healthcare Lifesciences, Uppsala, Sweden).

### In-gel digestion of proteins

The differentially abundant spots between the two extremes for RFI were cut out from the gels and placed in 1.5 mL tubes. Trypsinization was performed using a modified method based on Shevchenko et al. (2006). The gels pieces were destained through washes with a solution containing 50% acetonitrile (ACN) and 25 mM ammonium bicarbonate (Ambic), pH 8.0, and dried at room temperature. Subsequently, the solution was removed and samples were dehydrated in 100% ACN (200 μl). The reduction reaction was made with 100 μl of 65 mM DTT and 100 mM Ambic and alkylation was made with 100 μl of 200 mM iodoacetamide and 100 mM Ambic. For sample cleavage, 20 μl of Porcine Trypsin (Mass Spectrometry Grade, Promega, Madison, USA) was added to the fragments and kept on ice for 45 min, to allow trypsin to penetrate the gel fragments. Recovery of the tryptic peptides was made through the addition of a solution containing 5% formic acid and 50% ACN and removal of the supernatant. Samples were desalted using Zip Tip C18 micro columns (Millipore, Billerica, MA) to decrease interference from crystallization of the samples with the matrix used in a mass spectrometer analysis, and were then stored at − 20 °C until subsequent analysis.

### Protein identification

Peptide masses of the samples were obtained using an Ultraflex III MALDI TOF/TOF mass spectrometer (Bruker Daltonics, Bremen, Germany) and then screened against the Bovidae database deposited in UniProt using the MASCOT program (Matrix Science, London, UK) and a MASCOT Peptide Mass Fingerprinting database search. An accuracy of 0.5 Da was used in the search criteria. Trypsin was set as an enzyme with one allowed miscleavage. The fixed modification and variable modification factors used were carbamidomethyl and oxidation, respectively. The numbers of peptide matches, sequence coverage, molecular weight (MW), and *isoelectric point* (pI) were used to evaluate the database search results. The Scaffold program (Proteome Software, Portland, OR) was used to validate the proteins identified by the MASCOT program, with the identity for both proteins and peptides equal to 100%.

Protein abundance was quantified by spot densitometry analysis using ImageMaster 2D Platinum Software (GE Health Care, Little Chalfont-UK). The software used powerful algorithms for efficient spot detection, accurate spot quantification, and an accurate statistical comparison of spot density across gels.

### MicroRNA identification in silico

The identified proteins were used to search for potential microRNAs (miRNA). miRNAs were predicted using the web-based computational software TargetScan, which is freely available at http://www.targetscan.org. In summary, by default, this program identifies conserved sites of target genes that match the seed region of each miRNA [22]. Predictions were ranked based on the predicted efficacy of targeting based on cumulative weighted context++ scores of the sites, which combine multiple features to build quantitative models of targeting efficacy and thus indicates how efficiently a given miRNA may target on mRNAs according to Agarwal et al. [23]. The identified miRNAs ranked as the top three candidates for a post-transcriptional control of each protein found as differentially abundant between treatments were chosen to validate via reverse transcription quantitative polymerase chain reaction (RT-qPCR). In addition, aiming to double-check the miRNA prediction performed via TargetScan, a second web-based computational software, miRmap [24], was used. In summary, this software is based on thermodynamic, evolutionary, probabilistic and sequence-based approaches to predict potential targets providing a miRmap score. We also used the Cytoscape NetworkAnalyzer Tool [25] to illustrate the mRNA-miRNA interaction of the top three candidates’ miRNA. Illustrated interaction was based on context++ scores from the TargetScan program.

### Extraction of RNA enriched with miRNA and reverse transcription cDNA synthesis

Extraction of RNA enriched with miRNA was performed by using a *mir*Vana™ isolation kit (Invitrogen, California, USA) following the manufacturer’s instruction. The concentration of miRNA-enriched samples was estimated by a NanoVue™ Plus spectrophotometer (GE Healthcare, Freiburg, Germany) and their integrity was assessed through 1% agarose gel electrophoresis. The reverse transcription of total RNA-miRNA enriched samples was performed by using the miScript II RT kit (Qiagen, Hiden, Germany) following the manufacturer’s instruction. The abundance of both mRNA and miRNA was performed from the same cDNA sample to avoid possible bias due to differences in the extraction method.

### Quantitative real-time PCR analysis

Quantitative RT-PCR (qPCR) was performed in a thermal cycler ABIPrism 7300 Sequence Detection Systems (Applied Biosystems, Foster City, CA, USA) using an miScript SYBR Green PCR Kit (Qiagen, Hilden, Germany). Primers were obtained based on the mature sequence of the identified miRNAs in the present study (Table 2). Mature sequences were obtained in the miRBase database [26], according to each miRNA name. Primers used for the mRNA (Table 3) were selected based on their efficiency and specificity. The amplification efficiency ranged from 0.90 to 0.99. After amplification, a melting curve (0.01 °C/s) was used to confirm product purity. For the miRNA, due to its small size, the product of qPCR reaction was confirmed in 1% agarose gel. The qPCR reaction consisted of three cycle parameters: 95 °C for 3 min, 40 cycles at 95 °C for 10 s, and 60 °C for 30 s. The housekeeping gene used for the mRNA expression was the 18S, while the ncRNA-U6 was used for miRNA expression. No differences were observed (*P* > 0.05) for the expression of 18S and ncRNA-U6 between treatments. The expression of both mRNa and miRNA was calculated using the 2^-ΔCt^ method [27].

**Table 2:** Primers used to measure the relative abundance of miRNA using qRT-PCR

miRNA	Mature ID	Primer sequence (5′-3′)
bta-miR-2899	MIMAT0013857	AGGCGGGCCGGGGTTGGA
bta-miR-34a	MIMAT0004340	TGGCAGTGTCTTAGCTGGTTGT
bta-miR-449a	MIMAT0009320	TGGCAGTGTATTGTTAGCTGGT
bta-miR-665	MIMAT0009363	ACCAGTAGGCCGAGGCCCCT
bta-miR-2349	MIMAT0011884	TGGCACTTCTGGTCTCAGACTCA
bta-miR-3120	MIMAT0024572	CACAGCAAGTGTAGACAGGCA
bta-ncRNA U6	XR003033651	GTGCTCGCTTCGGCAGCAC

**Table 3:** Primers used to measure the relative abundance of mRNA using qRT-PCR

Gene Abbreviation^a^	Forward sequence (5′-3′)	Reverse sequence (3′-5′)	NCBI^b^
YWHAE	TCCCTCTGAAGCAGGTTAG	GGAGAGGGAAGGAGAAGAAA	NM_174491.3
HSPB1	CACTCGCAAATACACGCT	TGACGGGAATGGTGATCT	XM_005225115.2
18S	CCTGCGGCTTAATTTGACTC	AACTAAGAACGGCCATGCAC	NR_036642.1

^a^ YWHAE: Tyrosine 3-monooxygenase/tryptophan 5-monooxygenase activation protein; HSPB1: Heat Shock Protein family B member 1; 18S: ribossomal RNA

^b^ National Center for Biotechnology Information database (www.ncbi.nlm.nih.gov)

### Statistical analysis

The intensity of the normalized abundance volume of each spot obtained by two-dimensional electrophoresis, as well as mRNA and miRNA expression, were analyzed by using a linear model including the fixed effect of RFI group. After the initial analyses, the residuals from the analysis of each variable were assessed for normality using the Shapiro-Wilk’s test. The mRNA and miRNA expression data did not achieve normality, and it was transformed using Log(2^-ΔcT^). Least-squares means were estimated for the effect of RFI group. Results were deemed significant when *P* < 0.05. All analyses were performed using the MIXED procedure of SAS 9.4 (Statistical Analysis System Institute, Inc., Cary, NC, USA).

## Results

### Difference in protein abundance in skeletal muscle of RFI-high and RFI-low groups

Data from all nine animals from each experimental group were analyzed. The two dimensional proteomic analysis revealed proteins with molecular mass varying from 14.4 to 97 kDa and the isoelectric point ranging from 3 and 10. Thirteen spots were found with different in abundance (*P* < 0.05) in the skeletal muscle between the RFI-Low (more efficient) and RFI-High (less efficient) groups (Additional file 1: Figure S1). From the 13 spots that had different abundance in skeletal muscle tissue of RFI-Low and RFI-High groups, three were able to be identified via mass spectrometry with high accuracy (100% probability; Table 4). The failure to identify all the spots was due to the lack of a specific bovine protein databases that matched the data obtained in the present study and/or due to the percentage of protein identification probability lower than 100%. From the three spots identified in the skeletal muscle tissue, two had higher abundance in RFI-High animals (spots 1 and 2), which were identified as Actin Alpha 1 and 14-3-3 Protein Epsilon. One spot had a higher abundance in RFI-Low animals (spot 3) and it was identified as Heat Shock Protein Beta 1 (Table 4).

**Table 4:** Proteins identified in gels of skeletal muscle tissue from Low (−) and High (+) residual feed intake (RFI)

Spot^a^	Protein	UniProt accession number	Group of greater abundance	Mascot Score	% Protein identification probability^b^	% Protein Coverage^c^	Theoretical		Experimental		Matched peptides^d^
							MW	PI	MW	PI	
1	Actin, alpha 1, skeletal muscle OS=*Bos taurus* GN = ACTA1 PE = 2 SV = 1	A4IFM8_BOVIN	RFI-High	586	100%	18	42,051	5.23	42,943	5.27	5
2	14–3-3 protein epsilon OS=Bos taurus GN=YWHAE PE = 2 SV = 1	W5PRN8	RFI-High	174	100%	16	29,174	4.63	26,444	3.98	3
3	Heat shock protein beta-1 OS=Bos taurus GN=HSPB1 PE = 3 SV = 2	E1BEL7	RFI-Low	649	100%	44	22,393	5.98	24,205	5.58	7

^a^ Numbers shown in Additional file 1: Figure S1

^b^ Probability for validation by Scaffold of proteins identified by Mascot

^c^ Protein coverage calculated by Scaffold (identified amino acids/total amino acids)

^d^ Number of peptides identified in Mascot and validated by Scaffold

From the three proteins identified to be differentially abundant among RFI groups, we further investigated the 14-3-3 Protein Epsilon and Heat Shock Protein Beta 1 due to their possible involvement in energy expenditure in skeletal muscle, and thus, may have an important role in feed efficiency. Actin Alpha 1 is a structural protein of the skeletal muscle and had no direct role with energy expenditure process in this tissue; thus, it was not further investigated.

### MicroRNA identification

**Fig. 1:**
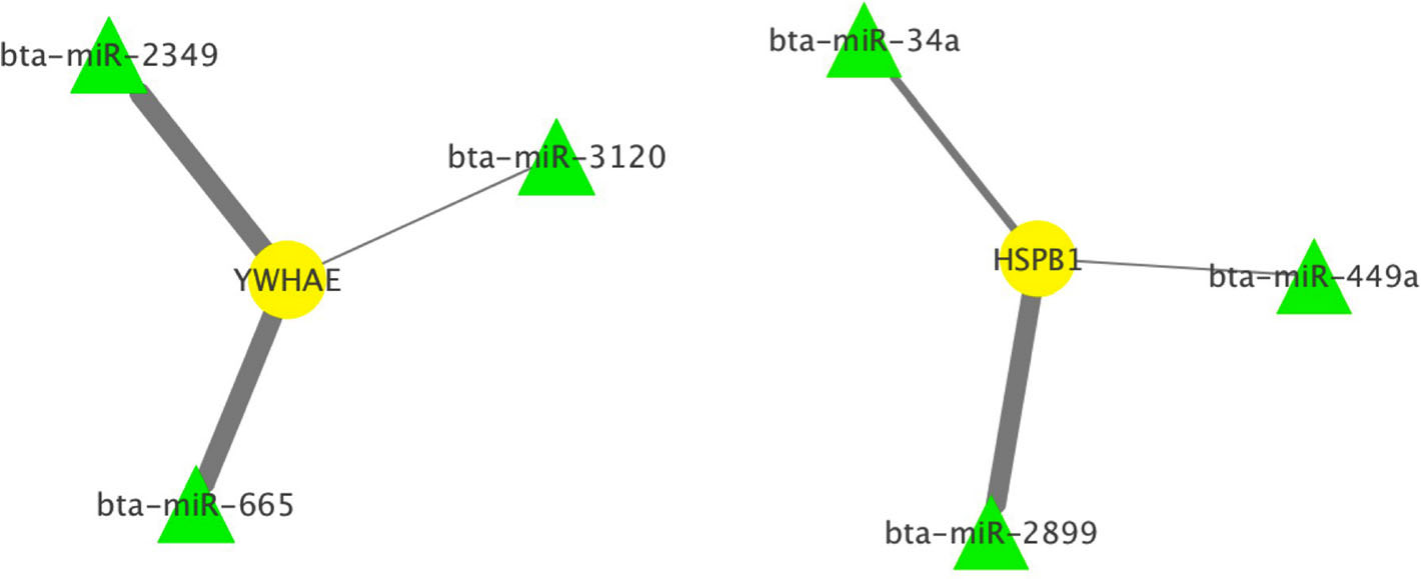
Highly predicted miRNAs associated with HSPB1 and YWHAE. Cytoscape NetworkAnalyzer Tool was used to illustrate mRNA-miRNA interaction. Interaction was based on context++ scores from TargetScan program. Thicker edges mean lower context++ scores and thus stronger interaction. The mRNAs are yellow circle nodes and miRNAs are green triangle nodes

Aiming to identify potential miRNAs related to differentially abundant proteins among RFI groups, we performed *in-silico* analyses based on 14-3-3 Protein Epsilon and Heat Shock Protein Beta 1 genes. For this aim, we used two web tools, TargetScan and miRmap, which identified 24 and 16 miRNAs for Heat Shock Protein Beta 1, respectively, and, 129 and 171 miRNAs for 14-3-3 Protein Epsilon, respectively (Additional file 2: Table S1, Additional file 3: Table S2). From these, comparing booth programs, six miRNAs were in common for Heat Shock Protein Beta and 90 were in common for 14-3-3 Protein Epsilon. Moreover, based on the TargetScan context++ scores, the top three miRNAs identified to match conserved sites of the two target genes were selected (Fig. 1).

### Difference in mRNA and miRNA involved in the synthesis of proteins the differentially abundant in skeletal muscle of RFI-high and RFI-low groups

Based on the results obtained from the proteomic study we further investigated the expression of miRNA in the skeletal muscle of both groups as a one of the possible mechanisms that led to changes in abundance of these proteins between the two groups. As miRNA acts post-transcriptionally, we also evaluated the expression of mRNA of genes that encodes the proteins that were found to be different in abundance between groups.

**Fig. 2:**
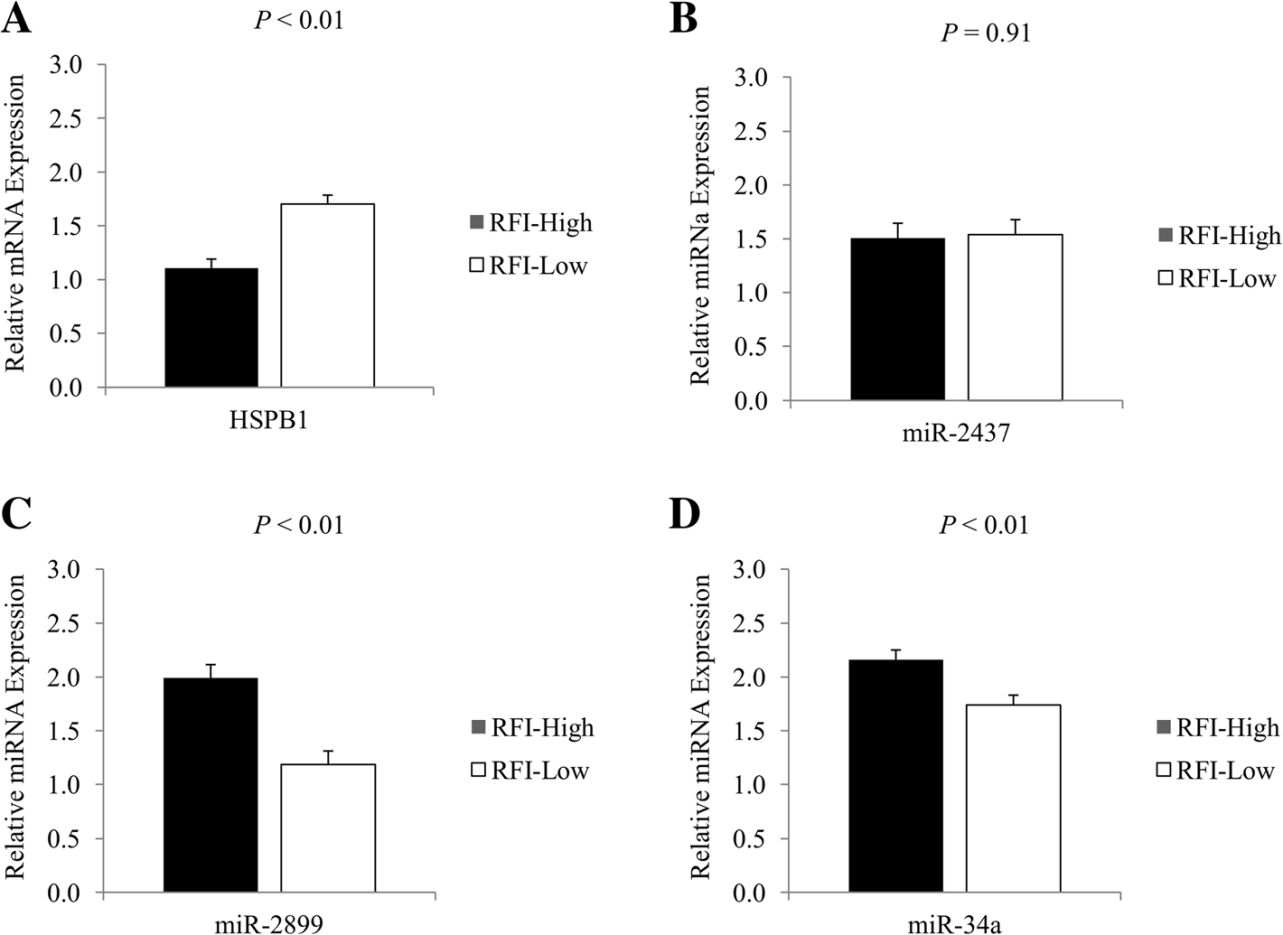
Relative expression of the gene that encodes Heat-Shock Protein β-1 and the miRNAs founded to be involved in the post-transcriptional control of its expression. **a** Relative expression (2^-ΔcT^) of *HSPB1* to *18S* in skeletal muscle of RFI-High and RFI-Low Nellore cattle (RFI-High = 1.13; RFI-Low = 3.79; SEM = 0.36); **b**) Relative expression (2^-ΔcT^) of miR-449a to ncRNA-U6 in skeletal muscle of RFI-High and RFI-Low Nellore cattle (RFI-High = 5.90; RFI-Low = 5.57; SEM = 0.73); **c**) Relative expression (2^-ΔcT^) of miR-2899 to ncRNA-U6 in skeletal muscle of RFI-High and RFI-Low Nellore cattle (RFI-High = 9.49; RFI-Low = 3.96; SEM = 1.05); **d**) Relative expression (2^-ΔcT^) of miR-34a to ncRNA-U6 in skeletal muscle of RFI-High and RFI-Low Nellore cattle (RFI-High = 6.71; RFI-Low = 2.81; SEM = 0.76). Differences were considered at *P* < 0.05

**Fig. 3:**
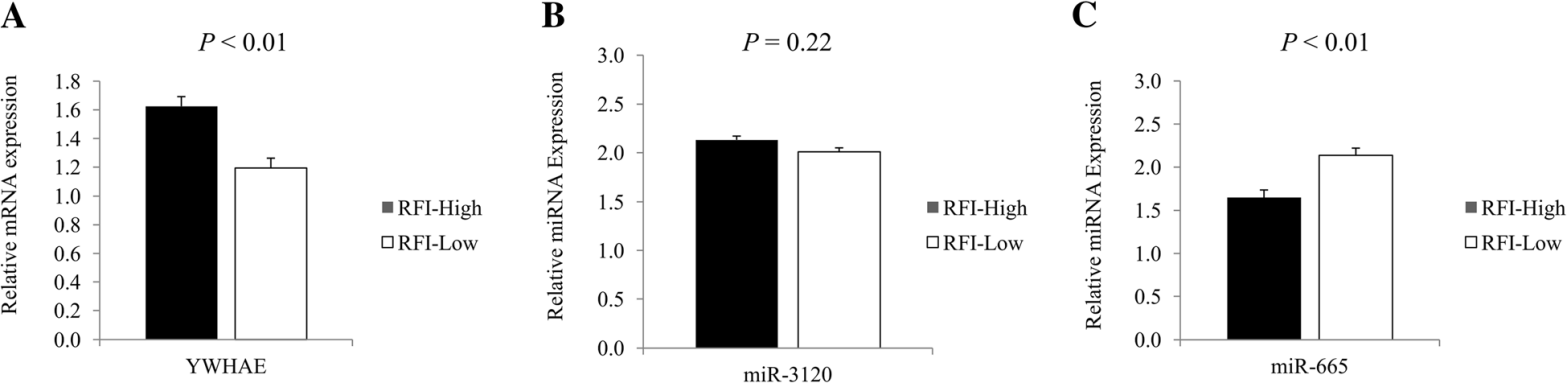
Expression of the gene that encodes 14–3-3 Protein Epsilon and the miRNAs founded to be involved in the post-transcriptional control of its expression. **a** Relative expression (2^-ΔcT^) of *YWHAE* to *18S* in skeletal muscle of RFI-High and RFI-Low Nellore cattle (RFI-High = 5.21; RFI-Low = 3.02; SEM = 0.35); **b**) Relative expression (2^-ΔcT^) of miR-3120 to ncRNA-U6 in skeletal muscle of RFI-High and RFI-Low Nellore cattle (RFI-High = 4.96; RFI-Low = 3.99; SEM = 0.59); **c**) Relative expression (2^-ΔcT^) of miR-665 to ncRNA-U6 in skeletal muscle of RFI-High and RFI-Low Nellore cattle (RFI-High = 4.94; RFI-Low = 10.44; SEM = 0.93). Differences were considered at *P* < 0.05

A greater mRNA expression of the *HSPB1* (*P* < 0.01), which is the gene that encodes the Heat-Shock Protein Beta 1, was observed in the skeletal muscle of RFI-Low group (Fig. 2). Conversely, a greater mRNA expression of *YWHAE*, which encodes the 14-3-3 Protein Epsilon, was observed in the RFI-High animals (*P* < 0.01; Fig. 3).

With regard to the miRNAs revealed by an *in-silico* study to be involved in the post-transcription control of the Heat-Shock Protein Beta 1, no differences were observed in the expression of miR-449a (*P* = 0.82) in the skeletal muscle of the RFI-High and RFI-Low groups (Fig. 2). On the other hand, a greater expression of miR-34a (*P* < 0.01) and miR-2899 (*P* < 0.01) was observed in the skeletal muscle of RFI-High group (*P* < 0.01; Fig. 2). For the top three miRNAs identified to be involved in the post-transcription control of 14-3-3 Protein Epsilon, only two of them were expressed in the skeletal muscle of the evaluated animals, whereas, no differences were observed for miR-3120 (*P* = 0.43) between the RFI-High and RFI-Low groups (Fig. 3). However, a greater expression of miR-665 was observed (*P* < 0.01) for RFI-Low compared to RFI-High animals (Fig. 3).

## Discussion

### Differences in abundance of proteins related to energy expenditure in skeletal muscle

The results of the current study revealed a greater abundance of Heat shock protein beta 1 (HSPB1), also known as HSP27, in RFI-Low compared to RFI-High animals. The HSPB1 is a relatively small molecular chaperone protein associated to cellular development, differentiation and signal transduction [28]. Heat shock proteins (HSPs) are essential for normal cellular stress responses [29] and may serve to protect cells from otherwise damaging agents [30]. Heat shock proteins are also able to bind and stabilize unstable proteins and facilitate their correct assembly. In addition, HSPs enhance cell survival by interfering with cellular signal transduction pathways regulating apoptotic cell death [31]. Small HSPs (sHSPs) bind unfolded polypeptides, acting as an important defense mechanism against the formation of protein aggregates [32]. This can reduce the binding of the proteases responsible for the degradation of muscle fibers [33]. Down-regulation of HSPB1 may increase the degradation of muscle proteins such as actin and myosin [34], which may increase the rate of protein turnover in skeletal muscle.

Previous studies have investigated the proteome profile in divergent RFI animals and have found that RFI selection cause changes in skeletal muscle protein turnover [35], mitochondrial protein profile [36] and changes in energy metabolism of skeletal muscle [37]. According to Cruzen et al. [35], animals selected for reduced RFI have less protein degradation. Such conclusion may be supported by a greater cytoprotective role of the HSPs proteins that were found to be greater in abundance in the skeletal muscle of more efficient animals, which was observed by Grubbs et al. [36] (HSP70) and also in our study (HSP27). The protein degradation is an energetically expensive process because of the required ATP for the proteasome operation [35]. Thus, the decrease of protein degradation in low-RFI animals suggested by the greater abundance of HSPs in their skeletal muscle may lead to a lower energy requirement for maintenance of this tissue, contributing to their greater feed-efficiency. Thus, our results suggest that because more efficient animals (RFI-Low) showed a greater abundance of HSPB1, they may have a lower rate of protein turnover and consequently lower energy expenditure in the skeletal muscle compared to less efficient animals (RFI-High), which may contribute to their difference in feed efficiency.

In the current study, we found a greater abundance of 14-3-3 epsilon protein in RFI-High (less efficient) than RFI-Low (more efficient) animals. The 14-3-3 proteins are small (~ 30 kDa) acidic proteins found in all eukaryotic organisms, with seven isoforms in mammals. All 14-3-3 s are highly conserved both within and across species [38] and have the ability to bind a large number of proteins causing multiple effects in specific proteins [39], including kinases, phosphatases, and transmembrane receptors. One of the functions of 14-3-3 epsilon is its interaction with the insulin-like growth factor I receptor (IGF-I R) and with the insulin receptor substrate I (IRS-1) [40]. A study performed by Oriente et al. [41] found that insulin action was enhanced following 14-3-3 epsilon overexpression and was reduced upon antisense depletion of 14-3-3 epsilon, and that overexpression of 14-3-3 epsilon also induced a reduction in insulin degradation. Previous studies have found no association between systemic insulin level and RFI in beef cattle [42, 43], which suggests that high and low RFI beef cattle do not differ in insulin production. The difference observed in the current study on 14-3-3 epsilon highlight the possible increase in insulin action that could lead to a higher uptake of insulin by skeletal muscle cells and increased glucose uptake. Therefore, based on the results observed for the HSPB1, it is plausible to suggest that such an event may occur in RFI-High animals due to a greater energy expenditure in their skeletal muscle as a consequence of a greater protein turnover, as previously stated. This possible mechanism was highlighted in a previous study, where HSPB1-null mice showed changes in the abundance of 14-3-3 epsilon in their skeletal muscle [44].

Another process that strongly contribute to energy expenditure in skeletal muscle is mitochondrial proton leakage [45], which is caused by the presence of uncoupling proteins (UCPs) located in the inner membrane of mitochondria. The UCPs cause the pumping of protons from the mitochondrial intermembrane space back to the mitochondrial matrix decreasing the electrochemical gradient necessary to generate ATP [46]. Despite the relevant importance of UCPs for energy metabolism in skeletal muscle, studies have shown similar or slight differences in the mRNA expression of UCP2 and UCP3 [12, 47] neither for UCP2 and UCP3 protein abundance in skeletal muscle [47] among high and low RFI beef cattle. Collectively, the lack of major changes in UCPs mRNA expression and the absence of these proteins in the proteomic results of the current study, as well as in the proteome of isolated skeletal muscle mitochondria [36] in skeletal muscle leads us to believe that, although UCP proteins may affect the energy expenditure in skeletal muscle at a cellular level, it does not differ in beef cattle selected for feed efficiency by RFI.

### Identification of miRNAs as possible post-transcriptional control of the differentially abundant proteins

MicroRNAs (miRNAs) have been reported as small noncoding RNA with a range from 18 to 23 nucleotides. The miRNAs act post-transcriptionally regulating gene expression by the target messenger (mRNA) in a sequence-specific manner, leading to either degradation of the target transcript and/or translational repression [48]. Studies have reported the association of miRNA and feed efficiency and energy metabolism in pigs [18], and cattle [14], showing the importance of the miRNA in the control of energy metabolism and subsequent changes in feed efficiency.

In a recent study designed to identify the possible role of miRNA in the regulation of RFI, a total of 156 mature miRNA sequences were identified in the skeletal muscle of beef cattle contrasting RFI. Of these, one miRNA (bta-miR-486) was differentially expressed between low and high RFI beef cattle [49]. The authors also reported the insulin pathway as a main target pathway of the bta-miR-486 in skeletal muscle being the protein kinase AMP-activated catalytic subunit alpha 1 (AMPK) in one of the targets. A study in pigs reported miRNAs associated with changes in energy metabolism between high and low RFI pigs [18]. Taken altogether, these studies highlight the involvement of miRNA in feed efficiency, likely by controlling energy utilization at the tissue level.

In our current study, after differential abundance of HSPB1 and 14-3-3 epsilon proteins in the skeletal muscle were identified between RFI-High and RFI-Low animals, we investigated why these difference may have occurred. We then evaluated the expression of candidate miRNAs that had possible involvement in the post-transcription modifications that resulted in changes in both proteins synthesis. As mentioned above, several studies have highlighted the importance of both proteins in the metabolism of skeletal muscle [44, 50–52]. However, to the best of our knowledge, this is the first study that investigated a possible molecular mechanism that may be responsible for the changes in the abundance of both proteins in skeletal muscle of beef cattle differently classified based on RFI.

Our results have shown a decrease of mRNA expression of *HSPB1* in skeletal muscle of RFI-High (less efficient) compared to RFI-Low (more efficient) animals. Such a result is supported by the greater expression of miR-34a and miR-2899 in the skeletal muscle of RFI-High animals, as both miRNAs have *HSPB1* transcript as a target. The miR-34a has been reported to be involved with apoptosis [53] and myoblast proliferation and differentiation [54], while miR-2899 was already identified to be expressed in beef cattle intramuscular and subcutaneous fat [55]. From our *in-silico* analyses, bta-miR-2899 had the greatest context++ score and can also be predicted by the TargetScan and miRmap programs. Based on these gene and miRNAs expressions, we may explain the differences in the abundance of HSPB1 protein between RFI-High and RFI-Low animals, as well as show a possible mechanism by which this protein biosynthesis is controlled in beef cattle differing in feed efficiency.

Another target mRNA that we have investigated in the current study was the *YWHAE*, which encodes the 14-3-3 protein epsilon. The mRNA expression of *YWHAE* was lower in the skeletal muscle of RFI-Low (more efficient) compared to RFI-High (less efficient) animals. This result may have occurred due to a greater expression of miR-665 in the skeletal muscle of RFI-Low animals. Such an observation likely explains the lower abundance of the 14-3-3 protein epsilon in RFI-Low compared to RFI-High animals. miR-665 has been reported to be differentially expressed during prenatal skeletal muscle development in pigs [56] and also expressed in cattle skeletal muscle [57]. This miRNA had the greatest context++ score (TargetScan) and one of the greatest miRmap scores, suggesting a strong interaction of *YWHAE* transcript. In our study, this miRNA is suggested to be associated with more feed efficient cattle.

The mechanisms of miRNAs controlling mRNA translation depend on the way miRNAs bind to their putative targets. A complete pairing (miRNA-mRNA) leads to a cleavage of the mRNA, while an imperfect binding might inhibit the translation [15, 58]. In our study, even though bta-miR-2899 and miR-665 were strongly linked with their respectively targets, the *in-silico* analyses suggest an imperfect pairing. Thus, based on our *in-silico* and in vitro analyses, it is expected that bta-miR-2899 and miR-665 may inhibit the translation of *HSPB1* and *YWHAE* transcripts, respectively.

It must be emphasized that miRNAs have several different targets. Thus, the results herein obtained may be taken into consideration not only for the control of the biosynthesis of the proteins that were found to be differentially abundant in the skeletal muscle of both RFI groups, but also for the contribution of the miRNAs in the control of the biological process underlying the discrepancy in feed efficiency. Moreover, to the best of our knowledge, this is the first time the miR-665, miR-34a, and miR-2899 were linked with feed efficiency in beef cattle.

## Conclusion

This study revealed differences in the abundance of HSPB1 and 14-3-3 epsilon proteins in skeletal muscle of Nellore beef cattle divergently identified for RFI. These results suggest that differences in energy expenditure in the skeletal muscle may contribute to their difference in feed efficiency. Moreover, we have shown differences in the mRNA expression of the genes encoding both proteins, which seems to be post-transcriptionally controlled by miRNA. Finally, our data also highlight the possible role of miR-34a, miR-2899, and miR-665 in the biological process underlying discrepancy in feed efficiency in beef cattle. These results warrant further studies to elucidate the mechanisms of control of these miRNAs in energy metabolism to our better understanding of biological process associated with feed efficiency.

## Additional files

Additional file 1: **Figure S1.** Representative 2-dimensional gel image of skeletal muscle from a Nellore bull. Proteins are indicated by spot number, which corresponds to those identified as differentially abundant between the different RFI groups. Spot 1: Actin, alpha 1, skeletal muscle; Spot 2: 14-3-3 protein epsilon; Spot 3: Heat shock protein beta-1. (TIF 372 kb)

Additional file 2: **Table S1.** Potential miRNAs related to Heat Shock Protein Beta 1 (HSPB1) identified via TargetScan and miRmap web tools. (DOCX 18 kb)

Additional file 3: **Table S2.** Potential miRNAs related to14-3-3 Protein Epsilon (YWHAE) identified via TargetScan and miRmap web tools. (DOCX 54 kb)

## Data Availability

All data is hosted at the Federal University of Viçosa-Brazil Repository and is freely available by contacting the corresponding author.

## Abbreviations

2-DE: Two-dimensional electrophoresis
ACN: Acetonitrile
ADG: Average daily gain
BSA: Bovine serum albumin
BW: Body weight
BW^0.75^: Metabolic body weight
cDNA: Complimentary DNA
DTT: Dithiothreitol
HSPB1: Heat-shock protein beta 1
IGF-I R: Insulin-like growth factor I receptor
IPG: Immobilized pH gradient
IRS-1: Insulin receptor substrate I
MADI - TOF: matrix-assisted laser desorption/ionization - time of flight
miRNA: micro RNA
mRNA: Messenger RNA
MW: Molecular weight
pI: Isoelectric point
PMSF: Phenylmethanesulfonyl fluoride
RFI: Residual feed intake
RT-qPCR: Reverse transcription quantitative polymerase chain reaction
SDS: Sodium dodecyl sulfate
sHSPs: Small heat-shock proteins
YWHAE: Gene that encodes the 14-3-3 protein epsilon
