# Supplementary figures and images for: Differentially expressed mRNAs, proteins and miRNAs associated to energy metabolism in skeletal muscle of beef cattle identified for low and high residual feed intake
Source: BMC Genomics. 2019 Jun 17;20:501. doi: 10.1186/s12864-019-5890-z (PMC6580615; doi:10.1186/s12864-019-5890-z)

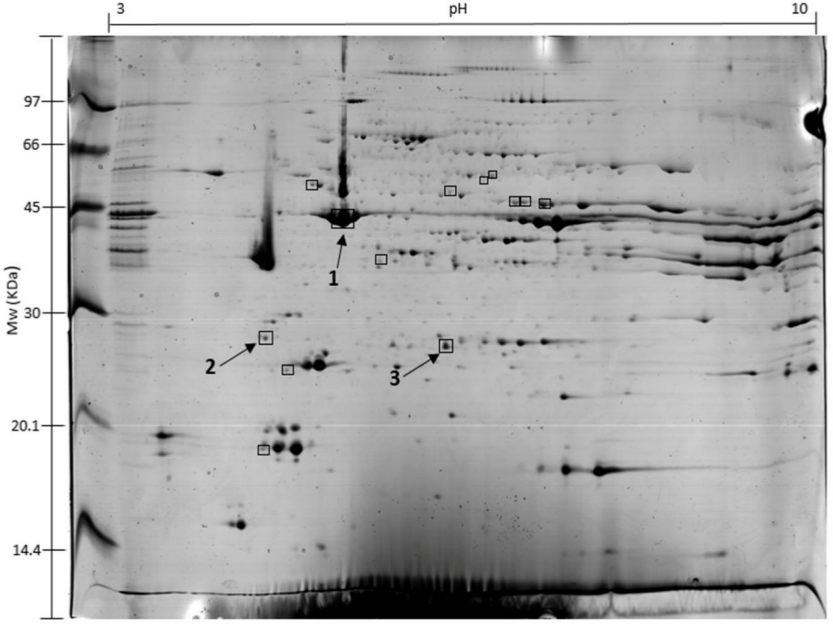

Supplement: Supplementary file 1 — Figure S1. Representative 2-dimensional gel image of skeletal muscle from a Nellore bull. Proteins are indicated by spot number, which corresponds to those identified as differentially abundant between the different RFI groups. Spot 1: Actin, alpha 1, skeletal muscle; Spot 2: 14-3-3 protein epsilon; Spot 3: Heat shock protein beta-1. (TIF 372 kb) [file 12864_2019_5890_MOESM1_ESM.tif]
