# Supplementary material for: Differentially expressed mRNAs, proteins and miRNAs associated to energy metabolism in skeletal muscle of beef cattle identified for low and high residual feed intake
Source: BMC Genomics. 2019 Jun 17;20:501. doi: 10.1186/s12864-019-5890-z (PMC6580615; doi:10.1186/s12864-019-5890-z)
Supplement: Supplementary file 2 — Table S1. Potential miRNAs related to Heat Shock Protein Beta 1 (HSPB1) identified via TargetScan and miRmap web tools. (DOCX 18 kb) [file 12864_2019_5890_MOESM2_ESM.docx]

Table S1. Potential miRNAs related to Heat Shock Protein Beta 1 (HSPB1) identified via TargetScan and miRmap web tools.

| **TargetScan** | | | | **miRmap** | | | | |
| --- | --- | --- | --- | --- | --- | --- | --- | --- |
| **miRNA** | **Position in the 3UTR** | **seed match** | **context++ score** | **miRNA** | **ΔG open** | **Probability exact** | **Conservation PhyloP** | **miRmap score** |
| bta-miR-2899 | 53-59 | 7mer-1A | -0.42 | bta-miR-329b | 98.86 | 98.60 | 0.00 | 88.65 |
| bta-miR-34a | 128-134 | 7mer-m8 | -0.40 | bta-miR-2322-3p | 97.72 | 97.36 | 1.02 | 84.23 |
| bta-miR-449a | 128-134 | 7mer-m8 | -0.39 | bta-miR-376e | 99.25 | 99.34 | 0.00 | 82.21 |
| bta-miR-449b | 128-134 | 7mer-m8 | -0.39 | bta-miR-2899 | 98.09 | 65.14 | 27.69 | 80.50 |
| bta-miR-34c | 128-134 | 7mer-m8 | -0.39 | bta-miR-877 | 99.25 | 93.47 | 0.00 | 75.74 |
| bta-miR-34b | 128-134 | 7mer-m8 | -0.39 | bta-miR-2309 | 97.38 | 76.93 | 1.99 | 72.04 |
| bta-miR-449c | 128-134 | 7mer-m8 | -0.38 | bta-miR-584 | 99.24 | 84.91 | 11.65 | 63.38 |
| bta-miR-760-3p | 136-142 | 7mer-m8 | -0.38 | bta-miR-186 | 99.36 | 85.33 | 80.59 | 51.25 |
| bta-miR-329b | 59-65 | 7mer-m8 | -0.37 | bta-miR-2386 | 98.87 | 84.91 | 11.65 | 47.37 |
| bta-miR-1842 | 136-142 | 7mer-m8 | -0.37 | bta-miR-223 | 98.86 | 95.91 | 52.90 | 36.75 |
| bta-miR-484 | 138-144 | 7mer-m8 | -0.36 | bta-miR-1185 | 98.30 | 90.71 | 1.99 | 24.39 |
| bta-miR-2450d | 41-47 | 7mer-m8 | -0.35 | bta-miR-135b | 98.92 | 79.69 | 46.42 | 24.23 |
| bta-miR-2450b | 41-47 | 7mer-m8 | -0.32 | bta-miR-135a | 98.92 | 79.69 | 46.42 | 20.32 |
| bta-miR-134 | 124-130 | 7mer-m8 | -0.31 | bta-miR-2450b | 98.51 | 97.74 | 37.24 | 86.56 |
| bta-miR-1185 | 46-52 | 7mer-1A | -0.30 | bta-miR-1224 | 97.72 | 87.11 | 1.99 | 53.36 |
| bta-miR-8550 | 137-143 | 7mer-m8 | -0.30 | bta-miR-2307 | 98.88 | 77.40 | 41.59 | 34.12 |
| bta-miR-2322-3p | 46-52 | 7mer-m8 | -0.28 |  |  |  |  |  |
| bta-miR-376e | 64-70 | 7mer-m8 | -0.28 |  |  |  |  |  |
| bta-miR-2440 | 127-133 | 7mer-m8 | -0.26 |  |  |  |  |  |
| bta-miR-2376 | 134-140 | 7mer-m8 | -0.24 |  |  |  |  |  |
| bta-miR-539 | 96-102 | 7mer-1A | -0.18 |  |  |  |  |  |
| bta-miR-205 | 152-158 | 7mer-1A | -0.16 |  |  |  |  |  |
| bta-miR-2285j | 143-149 | 7mer-m8 | -0.12 |  |  |  |  |  |
| bta-miR-2285u | 144-150 | 7mer-m8 | -0.05 |  |  |  |  |  |
