# Supplementary material for: Differentially expressed mRNAs, proteins and miRNAs associated to energy metabolism in skeletal muscle of beef cattle identified for low and high residual feed intake
Source: BMC Genomics. 2019 Jun 17;20:501. doi: 10.1186/s12864-019-5890-z (PMC6580615; doi:10.1186/s12864-019-5890-z)
Supplement: Supplementary file 3 — Table S2. Potential miRNAs related to14-3-3 Protein Epsilon (YWHAE) identified via TargetScan and miRmap web tools. (DOCX 54 kb) [file 12864_2019_5890_MOESM3_ESM.docx]

Table S2. Potential miRNAs related to14-3-3 Protein Epsilon (YWHAE) identified via TargetScan and miRmap web tools.

| **TargetScan** | | | | **miRmap** | | | | |  |
| --- | --- | --- | --- | --- | --- | --- | --- | --- | --- |
| **miRNA** | **Position in the 3'UTR** | **seed match** | **context++ score** | **miRNA** | **ΔG open** | **Probability exact** | **Conservation PhyloP** | **miRmap score** | |
| bta-miR-665 | 797-804 | 8mer | -0.53 | bta-miR-2360 | 69.32 | 68.78 | 44.93 | 98.30 | |
| bta-miR-2349 | 1153-1160 | 8mer | -0.53 | bta-miR-2449 | 69.68 | 68.78 | 44.93 | 97.41 | |
| bta-miR-3120 | 523-530 | 8mer | -0.46 | bta-miR-2467-3p | 69.29 | 63.12 | 33.32 | 97.21 | |
| bta-miR-877 | 1110-1117 | 8mer | -0.36 | bta-miR-2285j | 70.67 | 84.38 | 44.93 | 97.11 | |
| bta-miR-199a-3p | 796-802 | 7mer-m8 | -0.32 | bta-miR-665 | 45.61 | 94.91 | 44.93 | 96.71 | |
| bta-miR-2449 | 133-139 | 7mer-m8 | -0.32 | bta-miR-2393 | 45.85 | 12.72 | 20.43 | 9.66 | |
| bta-miR-2360 | 133-139 | 7mer-m8 | -0.32 | bta-miR-2881 | 64.62 | 94.56 | 0.00 | 95.85 | |
| bta-miR-3064 | 300-307 | 8mer | -0.32 | bta-miR-1777b | 63.99 | 94.55 | 14.12 | 95.53 | |
| bta-miR-493 | 42-48 | 7mer-m8 | -0.31 | bta-miR-423-5p | 84.22 | 83.41 | 25.74 | 94.99 | |
| bta-miR-135a | 339-345 | 7mer-m8 | -0.31 | bta-miR-744 | 54.19 | 96.85 | 0.00 | 94.66 | |
| bta-miR-135b | 339-345 | 7mer-m8 | -0.31 | bta-miR-2425-5p | 69.65 | 61.87 | 33.32 | 94.61 | |
| bta-miR-885 | 514-520 | 7mer-m8 | -0.31 | bta-miR-2900 | 63.92 | 94.56 | 0.00 | 94.45 | |
| bta-miR-22-5p | 1083-1090 | 8mer | -0.31 | bta-miR-135b | 72.51 | 73.25 | 22.51 | 92.68 | |
| bta-miR-653 | 545-552 | 8mer | -0.30 | bta-miR-6528 | 88.04 | 84.66 | 14.69 | 92.53 | |
| bta-miR-29b | 580-586 | 7mer-m8 | -0.30 | bta-miR-2882 | 70.27 | 97.57 | 0.00 | 92.41 | |
| bta-miR-182 | 903-909 | 7mer-1A | -0.30 | bta-miR-3578 | 95.10 | 80.79 | 44.93 | 92.14 | |
| bta-miR-142-3p | 849-856 | 8mer | -0.30 | bta-miR-2406 | 88.28 | 51.53 | 13.83 | 91.68 | |
| bta-miR-31 | 618-624 | 7mer-m8 | -0.29 | bta-miR-29d | 22.36 | 69.69 | 40.60 | 90.86 | |
| bta-miR-2398 | 37-43 | 7mer-m8 | -0.29 | bta-miR-29c | 22.36 | 69.69 | 40.60 | 90.77 | |
| bta-miR-29c | 580-586 | 7mer-m8 | -0.28 | bta-miR-2398 | 88.39 | 79.00 | 0.00 | 90.05 | |
| bta-miR-29d-3p | 580-586 | 7mer-m8 | -0.28 | bta-miR-2300b-3p | 39.08 | 70.95 | 44.93 | 89.72 | |
| bta-miR-1271 | 903-909 | 7mer-1A | -0.27 | bta-miR-2343 | 60.20 | 60.71 | 13.66 | 88.90 | |
| bta-miR-2887 | 217-223 | 7mer-1A | -0.27 | bta-miR-31 | 55.20 | 62.08 | 44.93 | 88.85 | |
| bta-miR-2369 | 888-894 | 7mer-1A | -0.27 | bta-miR-1814b | 37.04 | 71.44 | 40.60 | 8.82 | |
| bta-miR-22-5p | 991-997 | 7mer-m8 | -0.27 | bta-miR-199a-3p | 51.20 | 82.07 | 44.93 | 87.61 | |
| bta-miR-2425-5p | 135-141 | 7mer-m8 | -0.26 | bta-miR-3120 | 56.83 | 49.54 | 44.93 | 87.20 | |
| bta-miR-96 | 903-909 | 7mer-1A | -0.25 | bta-miR-767 | 25.98 | 83.29 | 40.60 | 87.05 | |
| bta-miR-744 | 726-732 | 7mer-m8 | -0.25 | bta-miR-493 | 89.27 | 77.58 | 0.00 | 86.77 | |
| bta-miR-3154 | 934-940 | 7mer-1A | -0.25 | bta-miR-2285h | 70.67 | 49.21 | 37.95 | 85.95 | |
| bta-miR-1843 | 783-789 | 7mer-1A | -0.23 | bta-miR-1343-5p | 76.39 | 83.41 | 12.01 | 84.08 | |
| bta-miR-151-3p | 954-960 | 7mer-1A | -0.23 | bta-miR-2285n | 70.67 | 49.21 | 37.95 | 83.29 | |
| bta-miR-200a | 1159-1165 | 7mer-m8 | -0.23 | bta-miR-2337 | 96.73 | 65.20 | 44.93 | 82.33 | |
| bta-miR-141 | 1159-1165 | 7mer-m8 | -0.23 | bta-miR-1306 | 49.14 | 57.70 | 33.27 | 81.88 | |
| bta-miR-6528 | 711-717 | 7mer-m8 | -0.22 | bta-miR-761 | 66.55 | 32.35 | 33.27 | 81.01 | |
| bta-miR-1256 | 901-907 | 7mer-1A | -0.22 | bta-miR-885 | 57.76 | 83.96 | 40.17 | 80.54 | |
| bta-miR-23a | 986-993 | 8mer | -0.22 | bta-miR-145 | 49.31 | 92.59 | 37.95 | 79.67 | |
| bta-miR-23b-3p | 986-993 | 8mer | -0.22 | bta-miR-2454-5p | 68.01 | 20.67 | 9.05 | 79.24 | |
| bta-miR-2480 | 1173-1179 | 7mer-m8 | -0.22 | bta-miR-2387 | 94.47 | 72.57 | 14.69 | 78.02 | |
| bta-miR-769 | 115-121 | 7mer-1A | -0.21 | bta-miR-494 | 75.06 | 51.33 | 20.43 | 77.98 | |
| bta-miR-1777b | 729-735 | 7mer-m8 | -0.21 | bta-miR-2287 | 39.71 | 38.53 | 37.95 | 76.39 | |
| bta-miR-2467-3p | 134-140 | 7mer-m8 | -0.20 | bta-miR-2382-5p | 76.39 | 83.41 | 12.01 | 75.99 | |
| bta-miR-2370-5p | 414-420 | 7mer-m8 | -0.20 | bta-miR-345-3p | 76.41 | 43.13 | 37.95 | 75.56 | |
| bta-miR-21-3p | 524-530 | 7mer-1A | -0.20 | bta-miR-2400 | 22.52 | 39.99 | 37.95 | 75.48 | |
| bta-miR-214 | 556-562 | 7mer-1A | -0.20 | bta-miR-380-3p | 78.82 | 89.28 | 6.65 | 75.33 | |
| bta-miR-767 | 579-585 | 7mer-m8 | -0.20 | bta-miR-185 | 77.18 | 90.74 | 20.43 | 75.16 | |
| bta-miR-2882 | 721-727 | 7mer-m8 | -0.20 | bta-miR-2887 | 39.71 | 49.57 | 37.95 | 74.80 | |
| bta-miR-3578 | 205-211 | 7mer-m8 | -0.19 | bta-miR-2447 | 35.57 | 35.93 | 37.95 | 73.03 | |
| bta-miR-2406 | 710-716 | 7mer-m8 | -0.19 | bta-miR-142-3p | 74.59 | 77.18 | 28.86 | 72.70 | |
| bta-miR-2386 | 905-911 | 7mer-1A | -0.19 | bta-miR-2347 | 64.68 | 41.82 | 33.27 | 72.37 | |
| bta-miR-494 | 1040-1046 | 7mer-m8 | -0.19 | bta-miR-2285a | 68.49 | 41.15 | 44.93 | 72.30 | |
| bta-miR-2418 | 50-56 | 7mer-1A | -0.18 | bta-miR-2341 | 49.11 | 37.49 | 20.43 | 72.18 | |
| bta-miR-2285a | 167-173 | 7mer-m8 | -0.18 | bta-miR-2334 | 66.55 | 32.35 | 33.27 | 72.15 | |
| bta-miR-761 | 556-562 | 7mer-1A | -0.18 | bta-miR-2450a | 69.49 | 31.45 | 37.95 | 72.04 | |
| bta-miR-95 | 655-661 | 7mer-1A | -0.18 | bta-miR-2479 | 99.63 | 25.87 | 27.65 | 71.68 | |
| bta-miR-2881 | 728-734 | 7mer-m8 | -0.18 | bta-miR-338 | 22.41 | 35.85 | 37.95 | 71.87 | |
| bta-miR-2900 | 728-734 | 7mer-m8 | -0.18 | bta-miR-214 | 66.55 | 32.35 | 33.27 | 70.96 | |
| bta-miR-6536 | 874-880 | 7mer-1A | -0.18 | bta-miR-769 | 49.78 | 59.53 | 37.95 | 70.70 | |
| bta-miR-412 | 989-995 | 7mer-1A | -0.18 | bta-miR-320b | 31.39 | 47.09 | 37.95 | 70.50 | |
| bta-miR-2370-5p | 1026-1032 | 7mer-1A | -0.18 | bta-miR-2284c | 63.60 | 26.46 | 20.43 | 6.88 | |
| bta-miR-873 | 130-136 | 7mer-m8 | -0.17 | bta-miR-2452 | 96.03 | 55.86 | 23.26 | 68.11 | |
| bta-miR-140 | 404-410 | 7mer-1A | -0.17 | bta-miR-370 | 39.49 | 63.87 | 33.68 | 65.82 | |
| bta-miR-2284b | 994-1000 | 7mer-m8 | -0.17 | bta-miR-2483-3p | 96.22 | 29.89 | 15.62 | 64.61 | |
| bta-miR-2284u | 994-1000 | 7mer-m8 | -0.17 | bta-miR-455-3p | 36.85 | 56.36 | 37.95 | 63.13 | |
| bta-miR-2337 | 20-26 | 7mer-m8 | -0.16 | bta-miR-2284d | 53.90 | 26.46 | 20.43 | 6.23 | |
| bta-miR-2285j | 143-149 | 7mer-m8 | -0.16 | bta-miR-2899 | 63.49 | 81.73 | 0.00 | 62.25 | |
| bta-miR-2461-5p | 192-198 | 7mer-1A | -0.16 | bta-miR-497 | 50.90 | 42.58 | 37.95 | 61.65 | |
| bta-miR-2300b-3p | 286-292 | 7mer-m8 | -0.16 | bta-miR-2411-3p | 73.90 | 43.13 | 37.95 | 61.55 | |
| bta-miR-2442 | 610-616 | 7mer-1A | -0.16 | bta-miR-21-3p | 65.69 | 20.58 | 37.95 | 61.00 | |
| bta-miR-2285g | 647-653 | 7mer-1A | -0.16 | bta-miR-140 | 55.12 | 23.72 | 5.22 | 60.91 | |
| bta-miR-2284y | 994-1000 | 7mer-m8 | -0.16 | bta-miR-2442 | 49.79 | 41.82 | 37.95 | 60.75 | |
| bta-miR-2284a | 994-1000 | 7mer-m8 | -0.16 | bta-miR-502b | 66.19 | 52.32 | 37.95 | 60.24 | |
| bta-miR-2284g | 994-1000 | 7mer-m8 | -0.16 | bta-miR-2354 | 50.45 | 37.49 | 20.43 | 59.94 | |
| bta-miR-2402 | 1120-1126 | 7mer-1A | -0.16 | bta-miR-6518 | 42.21 | 77.66 | 33.68 | 59.83 | |
| bta-miR-1388-3p | 84-90 | 7mer-1A | -0.15 | bta-miR-143 | 94.89 | 56.11 | 27.04 | 59.62 | |
| bta-miR-2334 | 556-562 | 7mer-1A | -0.15 | bta-miR-545-5p | 61.13 | 98.05 | 51.09 | 59.02 | |
| bta-miR-2285c | 647-653 | 7mer-1A | -0.15 | bta-miR-2370-5p | 56.27 | 30.20 | 0.00 | 58.73 | |
| bta-miR-2285d | 647-653 | 7mer-1A | -0.15 | bta-miR-149-3p | 54.07 | 22.44 | 14.69 | 58.58 | |
| bta-miR-2285e | 647-653 | 7mer-1A | -0.15 | bta-miR-2461-5p | 51.30 | 45.62 | 37.95 | 58.30 | |
| bta-miR-2343 | 735-741 | 7mer-m8 | -0.15 | bta-miR-15a | 49.80 | 42.58 | 37.95 | 57.51 | |
| bta-miR-2373-5p | 1049-1055 | 7mer-1A | -0.15 | bta-miR-224 | 45.95 | 15.66 | 37.95 | 56.62 | |
| bta-miR-155 | 273-279 | 7mer-m8 | -0.14 | bta-miR-2438 | 84.18 | 36.34 | 2.15 | 56.33 | |
| bta-miR-380-3p | 328-334 | 7mer-m8 | -0.14 | bta-miR-2284t-5p | 71.84 | 32.47 | 40.17 | 5.63 | |
| bta-miR-2284w | 551-557 | 7mer-m8 | -0.14 | bta-miR-2317 | 46.56 | 56.33 | 11.30 | 56.21 | |
| bta-miR-2488 | 596-602 | 7mer-1A | -0.14 | bta-miR-320a | 32.60 | 59.70 | 44.93 | 56.01 | |
| bta-miR-423-5p | 731-737 | 7mer-m8 | -0.14 | bta-miR-424-5p | 49.80 | 42.58 | 37.95 | 55.66 | |
| bta-miR-2393 | 945-951 | 7mer-m8 | -0.14 | bta-miR-1247-3p | 69.11 | 27.18 | 17.03 | 55.38 | |
| bta-miR-490 | 647-653 | 7mer-1A | -0.13 | bta-miR-15b | 50.90 | 42.58 | 37.95 | 55.38 | |
| bta-miR-7691 | 117-123 | 7mer-1A | -0.13 | bta-miR-495 | 73.09 | 71.90 | 37.95 | 53.60 | |
| bta-miR-7859 | 167-173 | 7mer-m8 | -0.13 | bta-miR-484 | 23.65 | 36.41 | 37.95 | 53.00 | |
| bta-miR-144 | 278-284 | 7mer-1A | -0.13 | bta-miR-3596 | 55.12 | 23.72 | 5.22 | 52.97 | |
| bta-miR-2483-3p | 471-477 | 7mer-1A | -0.13 | bta-miR-503-5p | 52.37 | 42.58 | 37.95 | 51.08 | |
| bta-miR-495 | 507-513 | 7mer-m8 | -0.13 | bta-miR-628 | 33.73 | 50.56 | 37.95 | 50.50 | |
| bta-miR-1814b | 598-604 | 7mer-m8 | -0.13 | bta-miR-6519 | 52.37 | 42.58 | 37.95 | 50.07 | |
| bta-miR-26b | 779-785 | 7mer-1A | -0.12 | bta-miR-16a | 50.90 | 42.58 | 37.95 | 49.09 | |
| bta-miR-26a | 779-785 | 7mer-1A | -0.12 | bta-miR-16b | 49.80 | 42.58 | 37.95 | 49.04 | |
| bta-miR-2285u | 144-150 | 7mer-m8 | -0.12 | bta-miR-875 | 57.16 | 57.73 | 0.00 | 48.60 | |
| bta-miR-199c | 278-284 | 7mer-1A | -0.12 | bta-miR-3604 | 80.99 | 6.97 | 5.51 | 47.08 | |
| bta-miR-2370-3p | 430-436 | 7mer-1A | -0.12 | bta-miR-155 | 30.91 | 82.81 | 44.93 | 46.91 | |
| bta-miR-2285j | 518-524 | 7mer-m8 | -0.12 | bta-miR-3431 | 73.68 | 44.19 | 32.82 | 46.50 | |
| bta-miR-2424 | 306-312 | 7mer-1A | -0.11 | bta-miR-2284w | 65.03 | 50.26 | 19.94 | 46.30 | |
| bta-miR-3596 | 404-410 | 7mer-1A | -0.11 | bta-miR-1721 | 40.80 | 31.99 | 23.26 | 46.13 | |
| bta-miR-545-5p | 844-850 | 7mer-1A | -0.11 | bta-miR-2424 | 55.84 | 44.24 | 27.04 | 45.76 | |
| bta-miR-7 | 1008-1014 | 7mer-1A | -0.11 | bta-miR-105a | 58.36 | 17.62 | 37.95 | 45.43 | |
| bta-miR-3431 | 83-89 | 7mer-1A | -0.1 | bta-miR-219-3p | 58.80 | 27.28 | 16.02 | 45.21 | |
| bta-miR-1721 | 831-837 | 7mer-1A | -0.1 | bta-miR-2437 | 51.86 | 34.44 | 37.95 | 44.99 | |
| bta-miR-2284ac | 866-872 | 7mer-1A | -0.1 | bta-miR-1434-3p | 88.32 | 1.74 | 4.96 | 4.42 | |
| bta-miR-320a | 222-228 | 7mer-m8 | -0.09 | bta-miR-2418 | 63.47 | 45.93 | 13.83 | 42.87 | |
| bta-miR-2284c | 103-109 | 7mer-1A | -0.09 | bta-miR-141 | 70.59 | 15.83 | 3.84 | 41.02 | |
| bta-miR-101 | 278-284 | 7mer-1A | -0.09 | bta-miR-105b | 58.36 | 17.62 | 37.95 | 40.25 | |
| bta-miR-6529a | 948-954 | 7mer-1A | -0.09 | bta-miR-541 | 83.33 | 36.50 | 11.20 | 40.22 | |
| bta-miR-2372 | 1146-1152 | 7mer-1A | -0.09 | bta-miR-2284j | 63.60 | 26.46 | 20.43 | 3.91 | |
| bta-miR-2311 | 766-772 | 7mer-1A | -0.08 | bta-miR-2299-3p | 79.81 | 81.71 | 0.00 | 38.35 | |
| bta-miR-2284t-5p | 453-460 | 8mer | -0.07 | bta-miR-2284n | 56.26 | 26.46 | 20.43 | 3.78 | |
| bta-miR-2957 | 778-784 | 7mer-1A | -0.07 | bta-miR-2284m | 63.60 | 26.46 | 20.43 | 3.68 | |
| bta-miR-2284ac | 908-914 | 7mer-1A | -0.07 | bta-miR-199c | 39.41 | 32.81 | 37.95 | 36.75 | |
| bta-miR-2284v | 103-109 | 7mer-1A | -0.06 | bta-miR-2486-3p | 55.24 | 29.38 | 37.95 | 35.67 | |
| bta-miR-3578 | 860-866 | 7mer-1A | -0.06 | bta-miR-329b | 40.82 | 26.88 | 37.95 | 35.05 | |
| bta-miR-545-5p | 211-217 | 7mer-m8 | -0.05 | bta-miR-2311 | 75.61 | 23.36 | 37.95 | 33.96 | |
| bta-miR-494 | 545-551 | 7mer-1A | -0.05 | bta-miR-200a | 62.24 | 15.83 | 38.43 | 32.99 | |
| bta-miR-181b | 984-990 | 7mer-m8 | -0.05 | bta-miR-582 | 28.73 | 39.11 | 37.95 | 32.28 | |
| bta-miR-181a | 984-990 | 7mer-m8 | -0.05 | bta-miR-1603 | 96.73 | 12.19 | 37.95 | 31.47 | |
| bta-miR-22-5p | 239-245 | 7mer-1A | -0.04 | bta-miR-2370-3p | 47.12 | 5.39 | 0.00 | 30.50 | |
| bta-miR-323 | 986-992 | 7mer-1A | -0.04 | bta-miR-2389 | 60.34 | 15.07 | 13.66 | 30.47 | |
| bta-miR-2284aa | 996-1002 | 7mer-m8 | -0.04 | bta-miR-490 | 52.25 | 33.96 | 37.95 | 29.66 | |
| bta-miR-2284z | 996-1002 | 7mer-m8 | -0.04 | bta-miR-1388-3p | 73.68 | 41.11 | 37.95 | 26.85 | |
| bta-miR-2284n | 103-109 | 7mer-1A | -0.03 | bta-miR-2396 | 23.20 | 40.46 | 37.95 | 26.81 | |
| bta-miR-2284ab | 103-109 | 7mer-1A | -0.03 | bta-miR-2957 | 78.29 | 10.80 | 37.95 | 26.54 | |
| bta-miR-2284d | 103-109 | 7mer-1A | -0.03 | bta-miR-26b | 78.26 | 20.93 | 37.95 | 26.24 | |
| bta-miR-181c | 984-990 | 7mer-m8 | -0.03 | bta-miR-144 | 28.74 | 32.81 | 37.95 | 26.06 | |
| bta-miR-181d | 984-990 | 7mer-m8 | -0.03 | bta-miR-26a | 78.26 | 20.93 | 37.95 | 25.80 | |
| bta-miR-186 | 693-699 | 7mer-m8 | -0.02 | bta-miR-205 | 32.07 | 32.04 | 37.95 | 25.43 | |
| bta-miR-3613b | 761-767 | 7mer-m8 | -0.02 | bta-miR-2480 | 79.86 | 43.40 | 0.00 | 23.04 | |
| bta-miR-2446 | 972-978 | 7mer-m8 | -0.02 | bta-miR-2488 | 35.90 | 30.86 | 33.68 | 22.69 | |
| bta-miR-2284k | 995-1001 | 7mer-m8 | -0.02 | bta-miR-219-5p | 32.29 | 44.00 | 33.68 | 21.60 | |
| bta-miR-2284m | 995-1001 | 7mer-m8 | -0.02 | bta-miR-2285c | 52.25 | 33.96 | 37.95 | 21.09 | |
| bta-miR-2284j | 995-1001 | 7mer-m8 | -0.02 | bta-miR-2285e | 52.25 | 33.96 | 37.95 | 21.02 | |
| bta-miR-2284ab | 995-1001 | 7mer-m8 | -0.02 | bta-miR-2285d | 52.25 | 33.96 | 37.95 | 20.76 | |
| bta-miR-2284n | 995-1001 | 7mer-m8 | -0.02 | bta-miR-380-5p | 36.64 | 29.12 | 37.95 | 20.66 | |
| bta-miR-2284d | 995-1001 | 7mer-m8 | -0.02 | bta-miR-375 | 99.44 | 7.03 | 27.65 | 20.17 | |
| bta-miR-2284v | 995-1001 | 7mer-m8 | -0.02 | bta-miR-2372 | 51.31 | 35.54 | 14.69 | 18.31 | |
| bta-miR-2284c | 995-1001 | 7mer-m8 | -0.02 | bta-miR-2301 | 96.73 | 12.19 | 37.95 | 18.04 | |
| bta-miR-2284k | 103-109 | 7mer-1A | -0.01 | bta-miR-2429 | 80.03 | 3.24 | 37.95 | 17.98 | |
| bta-miR-2284m | 103-109 | 7mer-1A | -0.01 | bta-miR-335 | 55.08 | 20.05 | 0.00 | 15.58 | |
| bta-miR-2284j | 103-109 | 7mer-1A | -0.01 | bta-miR-653 | 35.33 | 60.03 | 40.60 | 14.94 | |
| bta-miR-186 | 233-239 | 7mer-1A | -0.01 | bta-miR-2284v | 63.60 | 26.46 | 20.43 | 12.75 | |
| bta-miR-2284m | 626-632 | 7mer-1A | -0.01 | bta-miR-6529 | 94.72 | 38.44 | 27.04 | 11.75 | |
| bta-miR-2284k | 626-632 | 7mer-1A | -0.01 | bta-miR-2284z | 61.42 | 6.77 | 14.69 | 11.29 | |
| bta-miR-2284j | 626-632 | 7mer-1A | -0.01 | bta-miR-2284k | 56.26 | 26.46 | 20.43 | 10.10 | |
| bta-miR-2284ab | 626-632 | 7mer-1A | -0.01 | bta-miR-29b | 22.04 | 69.69 | 40.60 | 91.82 | |
| bta-miR-2284v | 626-632 | 7mer-1A | -0.01 | bta-miR-135a | 72.51 | 73.25 | 22.51 | 91.59 | |
| bta-miR-2284n | 626-632 | 7mer-1A | -0.01 | bta-miR-584 | 99.66 | 19.22 | 23.26 | 79.79 | |
| bta-miR-2284d | 626-632 | 7mer-1A | -0.01 | bta-miR-873 | 68.99 | 65.58 | 44.93 | 79.50 | |
| bta-miR-2284c | 626-632 | 7mer-1A | -0.01 | bta-miR-2369 | 99.66 | 26.34 | 23.26 | 67.22 | |
| bta-miR-2284t-5p | 1134-1140 | 7mer-1A | -0.01 | bta-miR-1843 | 77.45 | 38.10 | 37.95 | 61.38 | |
| bta-miR-2446 | 1139-1145 | 7mer-1A | -0.01 | bta-miR-186 | 58.92 | 38.25 | 37.95 | 5.31 | |
| bta-miR-2453 | 131-142 | non-canonical | N/A | bta-miR-543 | 66.51 | 48.63 | 37.95 | 52.68 | |
| bta-miR-2453 | 131-142 | non-canonical | N/A | bta-miR-2486-5p | 88.31 | 29.43 | 20.43 | 51.66 | |
| bta-miR-3613b | 997-1008 | non-canonical | N/A | bta-miR-6536 | 99.03 | 17.67 | 27.65 | 51.06 | |
| bta-miR-3613b | 997-1008 | non-canonical | N/A | bta-miR-195 | 50.90 | 42.58 | 37.95 | 47.19 | |
|  |  |  |  | bta-miR-95 | 38.90 | 72.81 | 0.00 | 44.01 | |
|  |  |  |  | bta-miR-2897 | 84.25 | 38.05 | 20.43 | 37.78 | |
|  |  |  |  | bta-miR-2297 | 54.43 | 29.37 | 37.95 | 29.44 | |
|  |  |  |  | bta-miR-22-5p | 20.68 | 25.84 | 0.00 | 27.56 | |
|  |  |  |  | bta-miR-101 | 29.55 | 32.81 | 37.95 | 27.01 | |
|  |  |  |  | bta-miR-2285g | 52.25 | 33.96 | 37.95 | 26.28 | |
|  |  |  |  | bta-miR-2285p | 68.49 | 3.24 | 37.95 | 17.23 | |
|  |  |  |  | bta-miR-2284aa-4 | 61.50 | 6.77 | 14.69 | 10.43 | |
|  |  |  |  | bta-miR-2284aa-3 | 61.50 | 6.77 | 14.69 | 10.43 | |
|  |  |  |  | bta-miR-2284aa-2 | 61.50 | 6.77 | 14.69 | 10.43 | |
|  |  |  |  | bta-miR-2284aa-1 | 61.50 | 6.77 | 14.69 | 10.43 | |
|  |  |  |  | bta-miR-2391 | 86.60 | 7.94 | 25.60 | 0.02 | |
